# Fetal programming: *in utero* exposure to acrylamide leads to intergenerational disrupted ovarian function and accelerated ovarian aging

**DOI:** 10.18632/aging.204269

**Published:** 2022-09-06

**Authors:** Nouf Aldawood, Maroua Jalouli, Abdulkarem Alrezaki, Saber Nahdi, Abdullah Alamri, Mohamed Alanazi, Salim Manoharadas, Saleh Alwasel, Abdel Halim Harrath

**Affiliations:** 1 Department of Zoology, College of Science, King Saud University, Riyadh, Saudi Arabia; 2 Department of Biochemistry, College of Science, King Saud University, Riyadh, Saudi Arabia; 3 Department of Botany and Microbiology, College of Science, King Saud University, Riyadh, Saudi Arabia

**Keywords:** acrylamide, transgeneration, apoptosis, female fertility, ovary aging

## Abstract

In this study we investigated the effects of multigenerational exposures to acrylamide (ACR) on ovarian function. Fifty-day-old Wistar albino female rats were divided into the control and ACR-treated groups (2.5, 10, and 20 mg/kg/day) from day 6 of pregnancy until delivery. The obtained females of the first (AF1) and second generation (AF2) were euthanized at 4 weeks of age, and plasma and ovary samples were collected. We found that *in utero* multigenerational exposure to ACR reduced fertility and ovarian function in AF1 through inducing histopathological changes as evidenced by the appearance of cysts and degenerating follicles, oocyte vacuolization, and pyknosis in granulosa cells. TMR red positive cells confirmed by TUNEL assay were mostly detected in the stroma of the treated groups. Estradiol and IGF-1 concentrations significantly decreased as a result of decreased *CYP19* gene and its protein expression. However, ACR exposure in AF2 led to early ovarian aging as evidenced by high estradiol and progesterone levels among all treated groups compared to control group, corresponding to the upregulation of the *CYP19* gene and protein expression. The apoptotic cells of the stroma were greatly detected compared to that in the control group, whereas no significant difference was reported in *ESR1* and *ESR2* gene expression. This study confirms the developmental adverse effects of ACR on ovarian function and fertility in at least two consecutive generations. It emphasizes the need for more effective strategies during pregnancy, such as eating healthy foods and avoiding consumption of ACR-rich products, including fried foods and coffee.

## INTRODUCTION

Maternal feeding before and during pregnancy can affect and modify fetal growth and some physiological parameters [1, 2], and healthy behavior during pregnancy and quality of food intake are positively related to the child’s height and weight [3, 4]. Thus, low birth weight can be considered as an indication of non-optimal prenatal development, which increases the risk of exposure to many diseases in later life, including type 2 diabetes and obesity [5–7]. Based on several reported studies, it is clear that any environmental disturbances have a harmful effect on fetal life, which subsequently leads to permanent diseases [8, 9]. Interestingly, clear connections between low exposure to the environment at the early stage of life and resulting occurrence of noncommunicable diseases, such as cardiovascular disease, obesity, diabetes, and cancer, and reproductive effects have been elaborated [10, 11]. For example, it is undoubtedly becoming apparent that exposure of female fetuses to excess androgen results in enlarged adult ovaries that are polyfollicular, anovulatory, and hyper androgenic and mimic those in polycystic ovary syndrome (PCOS) [12]. In contrast, high fetal estrogen level reduces ovarian size and function but increases adult anovulation [13–15]. This proves that the endocrine system during gestation closely controls the production of a wide variety of biological processes, and low-level exposure to endocrine-disrupting chemicals (EDCs) at these crucial windows of growth have the opportunity to alter vital incidents of enterprise [16]. Previous studies have found that prenatal exposure to certain organic compounds have adverse effects on the health of future generations. Particularly, prenatal susceptibility to xenobiotics, including environmental contaminants (e.g., smoke, sulfur dioxide, and carbon monoxide), medications (e.g., synthetic glucocorticoids), and categories of foods and drinks (e.g., ethanol and caffeine), could change the maternal status and/or harm the placenta, which indirectly affects fetal development. These xenobiotics will specifically cause irregular epigenetic modifications and expressions on vital fetal genes or disruption, which can also lead to the programming of fetal hypothalamic-pituitary axis modification [17]. The epigenetic modifications caused by prenatal exposure to xenobiotics for many generations may persist [18, 19]. Thus, exposures can be passed inheritably to future generations that have never been exposed to the compound. It has been suggested that this uncoded inheritability is induced by incomplete erasure during early pregnancy [20]. While the transmission is probable since imprinted epigenetic information is involved in this process, it is still to be identified [21, 22].

In contrast, millions of people worldwide have infertility as a result of reproductive disorders [23]. In both men and women, developmental exposure to EDC may have harmful effects to reproductive health. However, our understanding of the negative effects of chemicals on health in women is less than those in men [24]. Based on the formation of ACR in food during high temperatures and its presence in water and cosmetics [25, 26], this potential EDC may constitute a major problem for human health and could notably affect female fertility by influencing the ovary structure and function. Although understanding the effect of ACR on reproduction is important from both the theoretical and practical viewpoint, the mechanisms of its effects on different generations are still unknown [27–31]. Therefore, the present study aimed to investigate ovarian developmental toxicity on two generations of rats induced by ACR effect. We tried to observe the morphological, histopathological, and hormonal changes in the ovaries of the first and second generations. Furthermore, we explored the ovarian apoptotic cell death level and levels of expression of some key genes and proteins involved in the regulation of the folliculogenesis and steroidogenesis.

## RESULTS

### Effect of acrylamide on ovary weight (ovary index)

**Figure 1:**
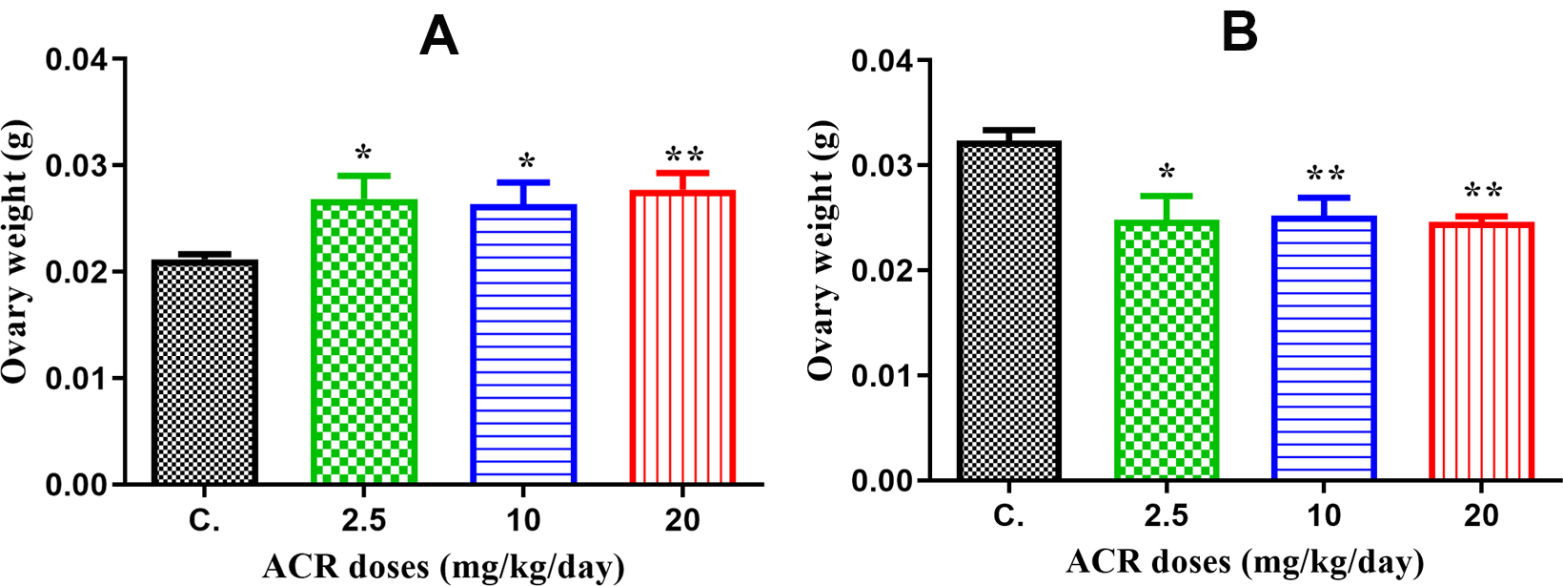
(**A**) The ovary weight in AF1 groups compared to that in the control group CF1 recorded a significant elevation with all doses of ACR (2.5, 10, and 20 mg/kg/day). (**B**) The ovary weight in AF2 groups recorded a significant decrease in ovary weight with all doses compared to that in the control group CF2.

The ovary weight significantly increased with all doses in the AF1 groups compared to that in the control group CF1 (Figure 1A), whereas the ovary weight of AF2 females significantly decreased with all treatments (Figure 1B).

### Effect of acrylamide on ovarian histopathological changes

**Figure 2:**
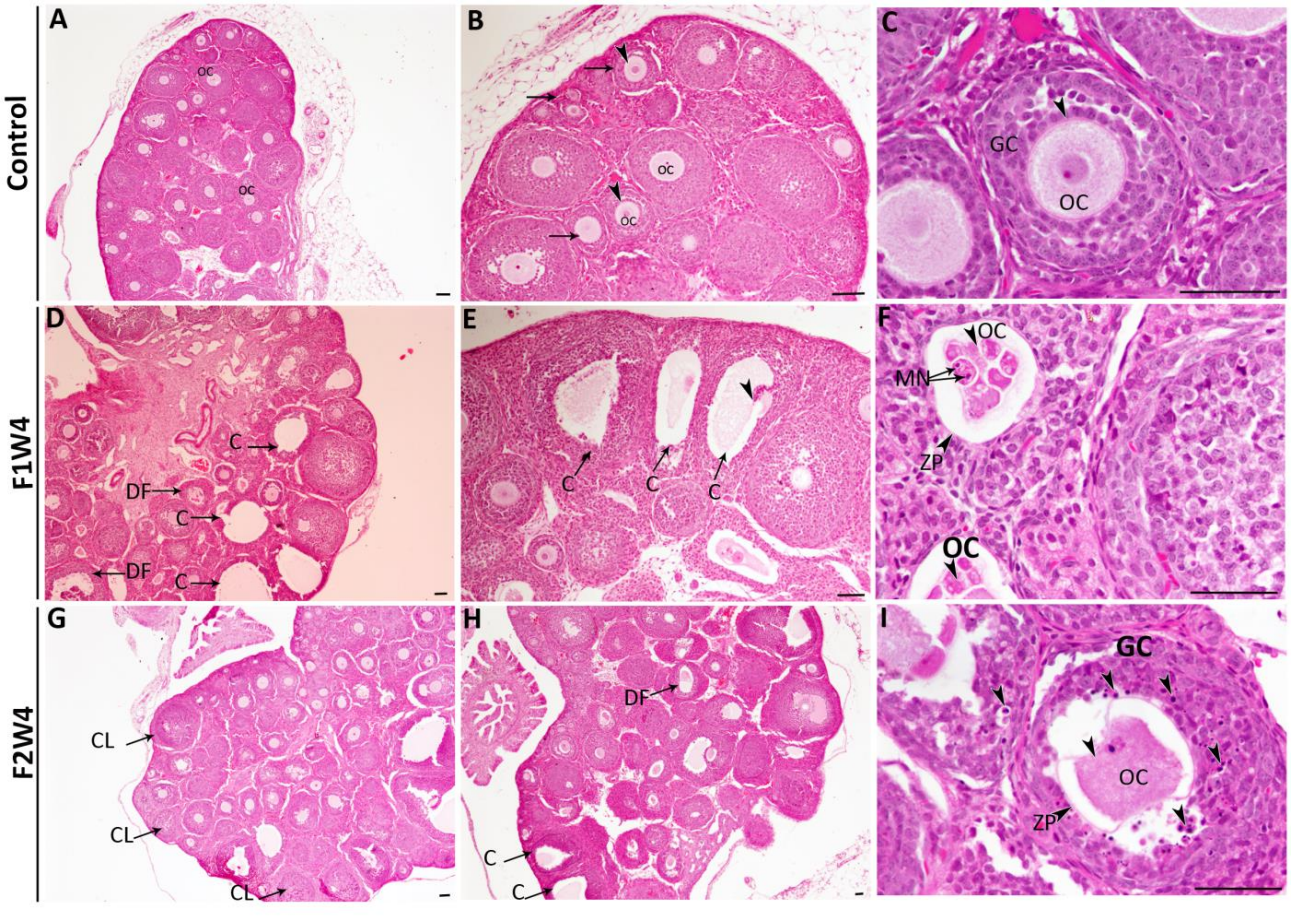
**Histopathological changes in the ovaries from the first (F1W4) and the second generation (F2W4) compared to those in the control group (stained with H&E).** (**A**–**C**) Photomicrographs at different magnifications of the same ovarian tissue section in females of control (CF1) showed normal structure containing normal growing follicles (arrows), normal oocytes (OC) (arrowheads), and a diminished number of pyknotic nuclei in granulosa cells (GC). (**D**, **E**) Photomicrographs of ovarian sections in treated females of F1W4 showing the presence of cysts (C), degenerated follicles (DF). (**F**) Segmented oocytes (arrowheads) enclosed by an irregular zona pellucida (ZP) with formation of micronucleus (MN). (**G**, **H**) Photomicrographs of ovarian sections in treated females of F2W4 showing a significant increase in growing follicle number and presence of corpora lutea (CL), there were a number of cysts (C) (arrows) and altered oocytes enclosed with abnormal zona pellucida (ZP), (**I**) in addition an elevation of pyknotic nuclei in granulosa cells (GC) (arrowheads). Scale bar = 60 μm.

Ovarian sections from 4-week-old females from the control group showed a normal structure of the ovary, which contained a large number of growing follicles at various stages of maturity (Figure 2A–2C). However, AF1 females that were treated with ACR showed the appearance of cysts and degenerating follicles (Figure 2D, 2E). The ooplasm sometimes appear segmented, and each segment contains fragments of highly compacted nuclei with formation of micronucleus (Figure 2F). Histological ovarian sections from females of the second generation (AF2) showed a high number of corpora lutea as evidenced by early sexual maturity compared to the control group (Figure 2G, 2H). Most granulosa cells have a high number of pyknotic nuclei and seem detached from the oocyte (Figure 2I). The deterioration and damage in the oocyte detected in most developing follicles were accentuated.

### Effect of acrylamide on progesterone, estradiol, testosterone and IGF-1 release

**Figure 3:**
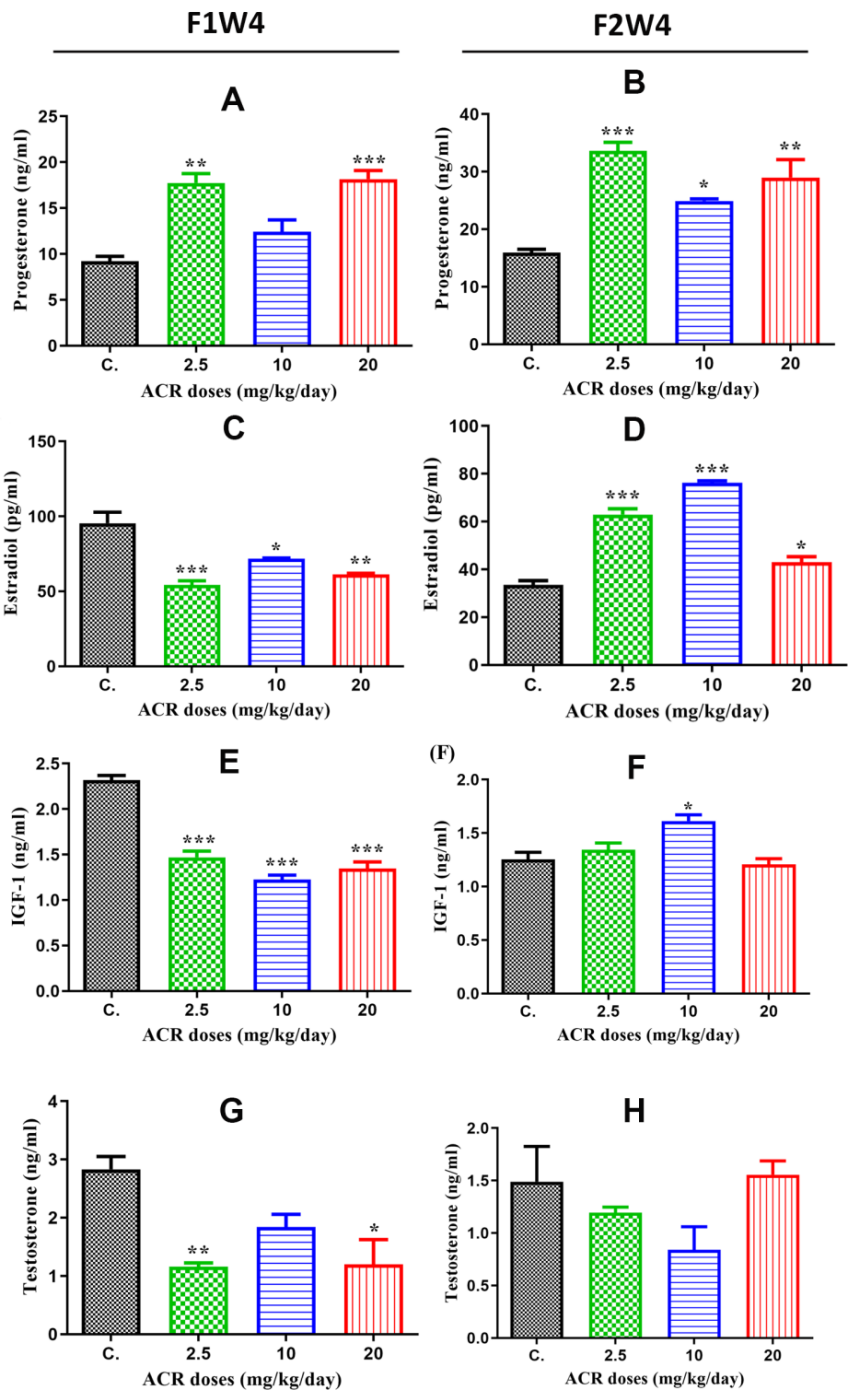
**Hormone levels in the plasma of female offspring rats exposed to ACR during their fetal life compared to those in controls.** (**A**) Plasma progesterone levels (ng/mL) in AF1 significantly increased in 2.5 and 20 mg/kg/day doses compared to the control group, while the increase in 10 mg/kg/day dose was nonsignificant. (**B**) Plasma progesterone levels (ng/mL) in AF2 were significantly elevated in all ACR dose (2.5, 10, and 20 mg/kg/day) groups compared to that in the control group CF2. (**C**) Plasma estradiol levels in AF1 groups recorded a high significant decrease in all doses of ACR. (**D**) A Plasma estradiol levels significantly increased in all AF2 treated groups compared to the control group CF2. (**E**) Plasma IGF-1 levels significantly decreased in all treated groups of AF1 compared to the control group. (**F**) We found a significant increase in plasma IGF-1 levels in the AF2 group treated with 10 mg/kg/day, whereas no significant variation was found in 2.5 and 20 mg/kg/day groups compared to the control group CF2. (**G**) Plasma testosterone levels in AF1 groups show a significant decline in 2.5 mg/kg/day and 20 mg/kg/day, whereas no significant variation was noted in 10 mg/kg/day. (**H**) No significant changes were observed in plasma testosterone levels in all AF2 treated groups compared to the control group CF2.

The plasma progesterone, estradiol, testosterone, and IGF-1 levels from different groups of rats are presented in Figure 3. plasma progesterone levels were significantly increased in AF1 females with both doses of 2.5 and 20 mg/kg/day compared to those in control CF1 (P < 0.05), while no significant difference was observed in the treatment with the dose of 10 mg/kg/day (Figure 3A). Similarly, progesterone levels significantly increased in AF2 groups with all doses of ACR treatment (2.5, 10, and 20 mg/kg/day) compared to that in the control CF2 group (Figure 3B).

A statistically significant decrease in plasma estradiol level was observed in all groups of treated females in AF1 compared to that in the control group (Figure 3C), while the estradiol level in AF2 significantly increased with all ACR doses of treatment (2.5 and 10 mg/kg/day) (Figure 3D). The results of plasma IGF-1 levels were similar to that of estradiol since this hormone was significantly decreased in all treated groups of AF1 compared to that in the control group (Figure 3E). However, only the dose of 10 mg/kg/day increased IGF-1 level in AF2 females compared to that in the control group CF2 (Figure 3F). Plasma testosterone levels were significantly decreased in AF1 females treated with doses of 2.5 mg/kg/day and 20 mg/kg/day (Figure 3G), while no significant variation was observed in all groups of AF2 females (Figure 3H).

### Effect of acrylamide on CYP 19 and GDF-9 protein expression

### Protein CYP19 (aromatase)

**Figure 4:**
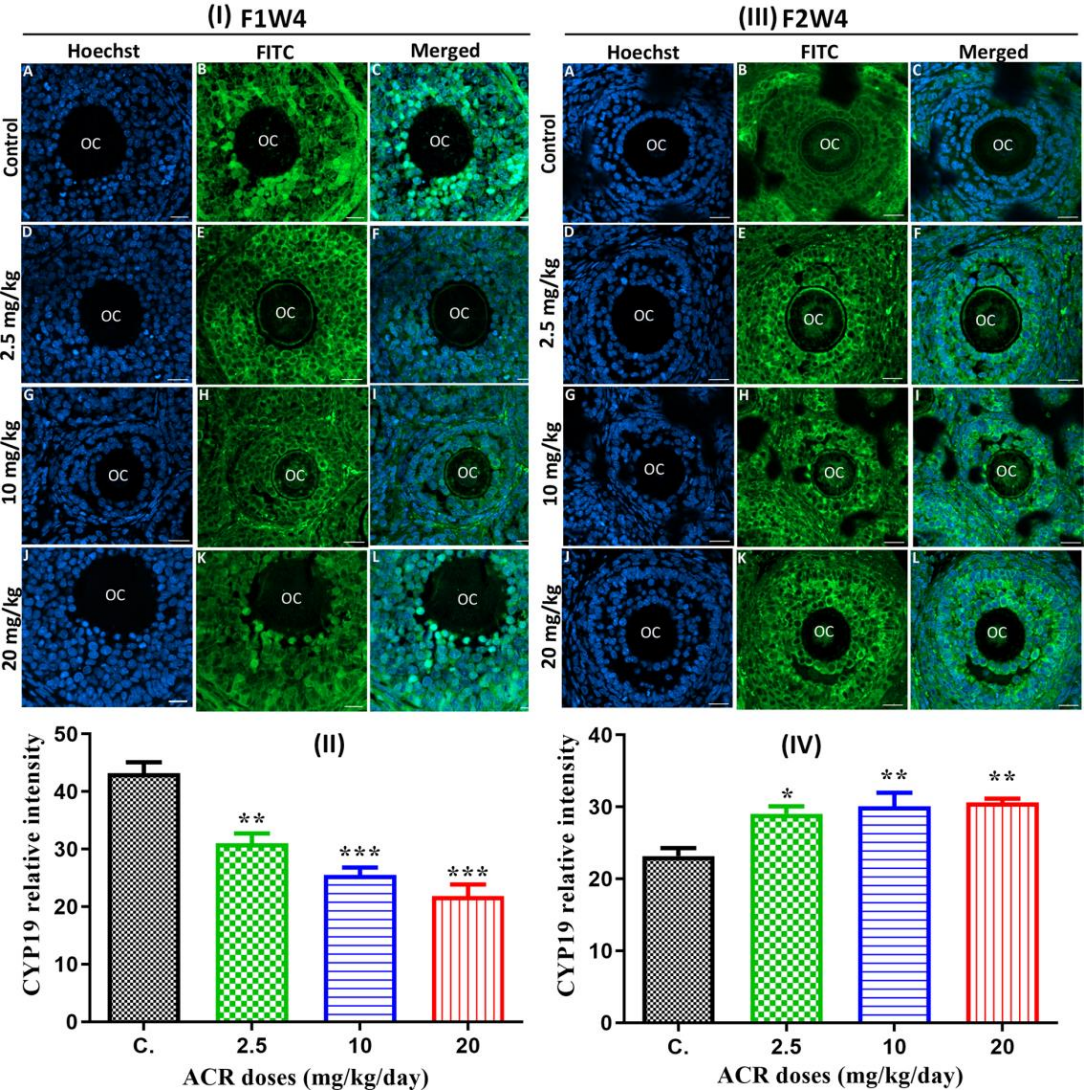
(**I**) Effect of developmental ACR exposure on immunolocalization of ovarian CYP19 localization in AF1 treated groups compared to control CF1. Normal control CF1 females (**A**–**C**). Immunolocalization of CYP19 protein in AF1 females treated with the dose of 2.5 mg/kg (**D**–**F**), 10 mg/kg (**G**–**I**), and 20 mg/kg (**J**–**L**). (**II**) CYP19 relative intensity in 4-week-old AF1 females compared with control CF1. (**III**) Effect of developmental ACR exposure on immunolocalization of ovarian CYP19 localization in AF2 treated groups compared to control CF2. The normal control CF2 females (**A**–**C**), immunolocalization of CYP19 protein in AF2 females treated with the dose of 2.5 mg/kg (**D**–**F**), 10 mg/kg (**G**–**I**)), and 20 mg/kg (**J**–**L**). (**IV**) The CYP19 relative intensity in 4-week-old AF2 females compared with control (CF2). Sections of the ovary was performed by immunofluorescence using specific CYP19 antibody stained with FITC (green), and cell nuclei were stained with Hoechst (blue). Scale bar = 20 μm.

The confocal microscopy images showed that green fluorescence intensity of CYP19 was significantly reduced (P < 0.05) in ovaries of AF1 females exposed to the different ACR doses (Figure 4I, 4II) and compared to untreated control females. However, the results showed that the protein expression level of CYP19 was significantly increased (P < 0.05) in ovaries of AF2 rats exposed to the different doses of ACR compared to untreated control rat AC2 (Figure 4III, 4IV).

### Protein GDF-9

**Figure 5:**
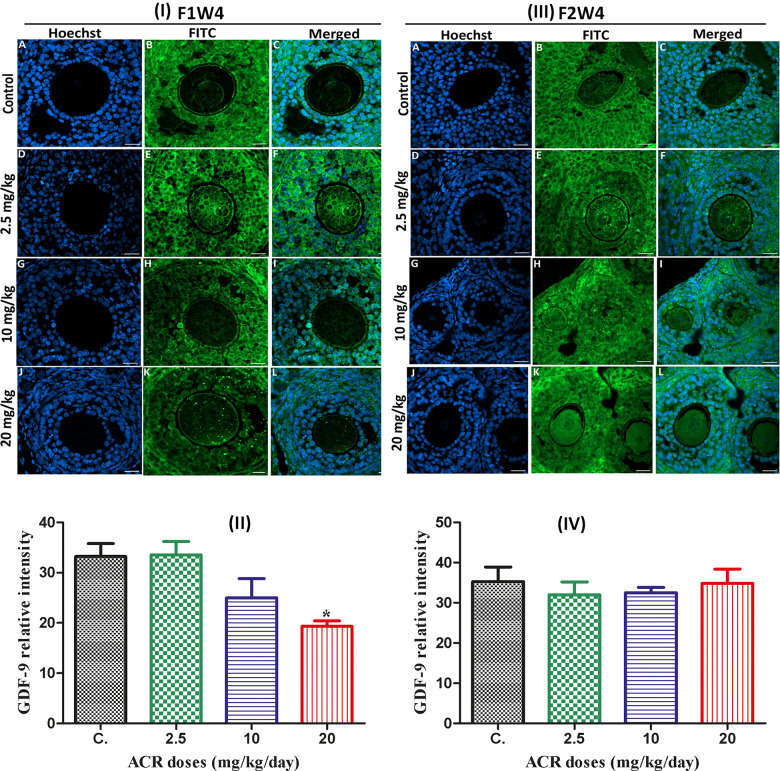
(**I**) Effect of developmental ACR exposure on immunolocalization of ovarian GDF-9 localization in AF1 treated groups compared to control CF1. Normal control CF1 females (**A**–**C**). Immunolocalization of GDF-9 protein in AF1 females treated with the dose of 2.5 mg/kg (**D**–**F**), 10 mg/kg (**G**–**I**), and 20 mg/kg (**J**–**L**). (**II**) CYP19 relative intensity in 4-week-old AF1 females compared with control CF1. (**III**) Effect of developmental ACR exposure on immunolocalization of ovarian GDF-9 localization in AF2 treated groups compared to control CF2. The normal control CF2 females (**A**–**C**), immunolocalization of GDF-9 protein in AF2 females treated with the dose of 2.5 mg/kg (**D**–**F**), 10 mg/kg (**G**–**I**)), and 20 mg/kg (**J**–**L**). (**IV**) The GDF-9 relative intensity in 4-week-old AF2 females compared with control (CF2). Sections of the ovary was performed by immunofluorescence using specific GDF-9 antibody stained with FITC (green), and cell nuclei were stained with Hoechst (blue). Scale bar = 20 μm.

The results showed that green fluorescence intensity of GDF-9 in ovaries from treated AF1 females was similar to that in control group CF1, except the dose of 20 mg/kg/day, in which the signal was weaker than that in the control group (Figures 5I, 4II). With increasing ACR doses, no significant difference in the GDF-9 protein expression was noted between the ACR-treated females of the AF2 and control groups (Figure 4III, 4IV).

### Effect of acrylamide on ovarian cell apoptosis

**Figure 6:**
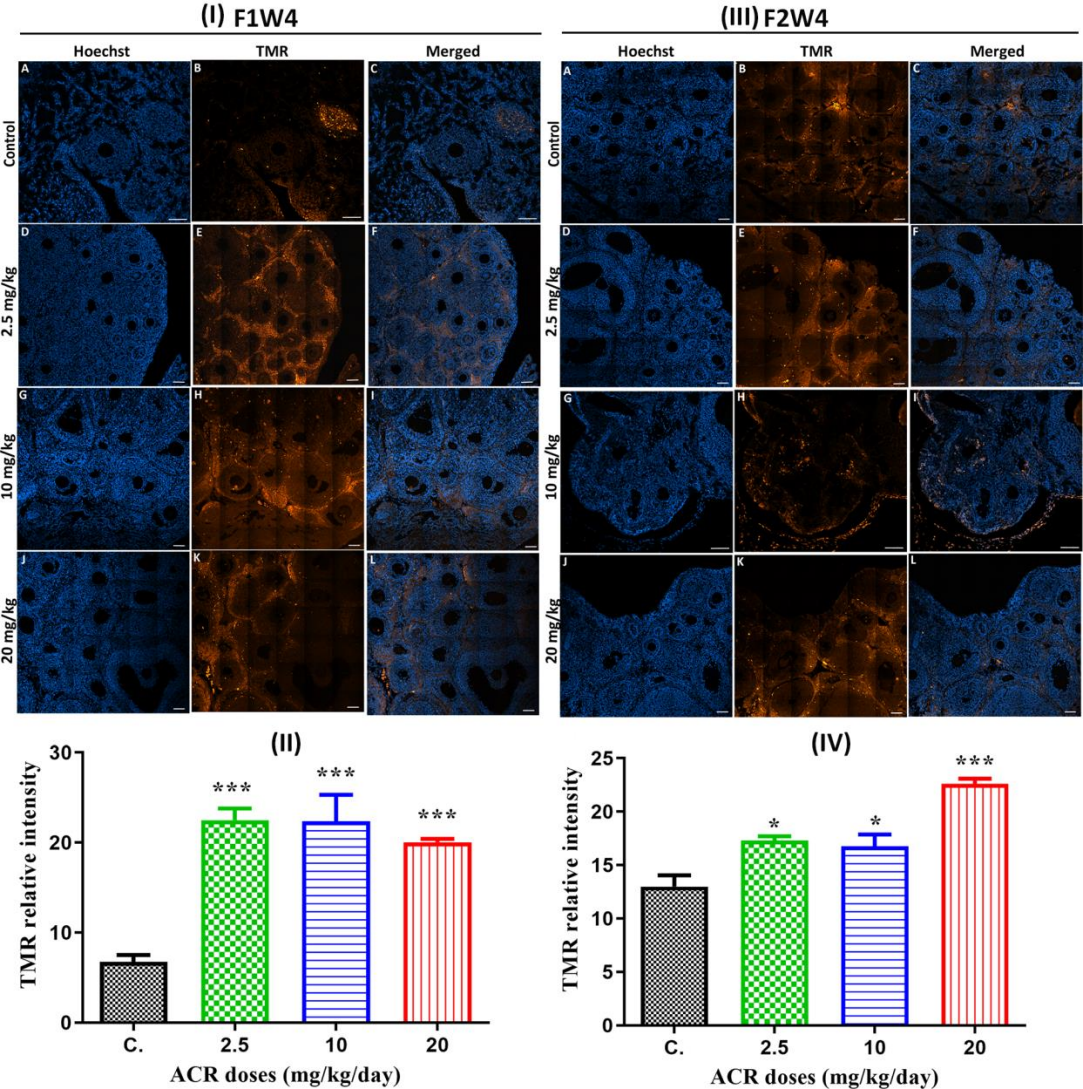
(**I**) TUNEL staining (red) of ovaries from AF1 females and normal CF2 females (**A**–**C**), and ovaries from AF1 with the dose of 2.5 mg/kg (**D**–**F**), 10 mg/kg (**G**–**I**), and 20 mg/kg (**J**–**L**). Both granulosa cells and oocyte nuclei were stained with Hoechst (blue). (**II**) TMR relative intensity in 4-week-old AF1 females compared with control CF1. (**III**) TUNEL staining (red) of ovaries from AF2 females and normal CF2 females (**A**–**C**), and ovaries from AF1 with the dose of 2.5 mg/kg (**D**–**F**), 10 mg/kg (**G**–**I**), and 20 mg/kg (**J**–**L**). Both granulosa cells and oocyte nuclei were stained with Hoechst (blue). (**IV**) TMR relative intensity in 4-week-old AF2 females compared with control CF2. Scale bar = 60μm.

A significant increase in the TUNEL positive cells was observed in the ovarian sections of all 4-week-old females of the first generation (AF1) when treated with the different doses (Figure 6I, 6II). Similarly, TUNEL-positive cells were also detected in the ovarian sections of AF2 females from the different treated groups compared to control (Figure 6III, 6IV). The TUNEL positive cells were mainly detected in the stroma cells, whereas almost no significant signal was detected in theca and granulosa cells.

### Effect of acrylamide on CYP 19, ESR1, and ESR2 gene expression

### CYP19 gene

**Figure 7:**
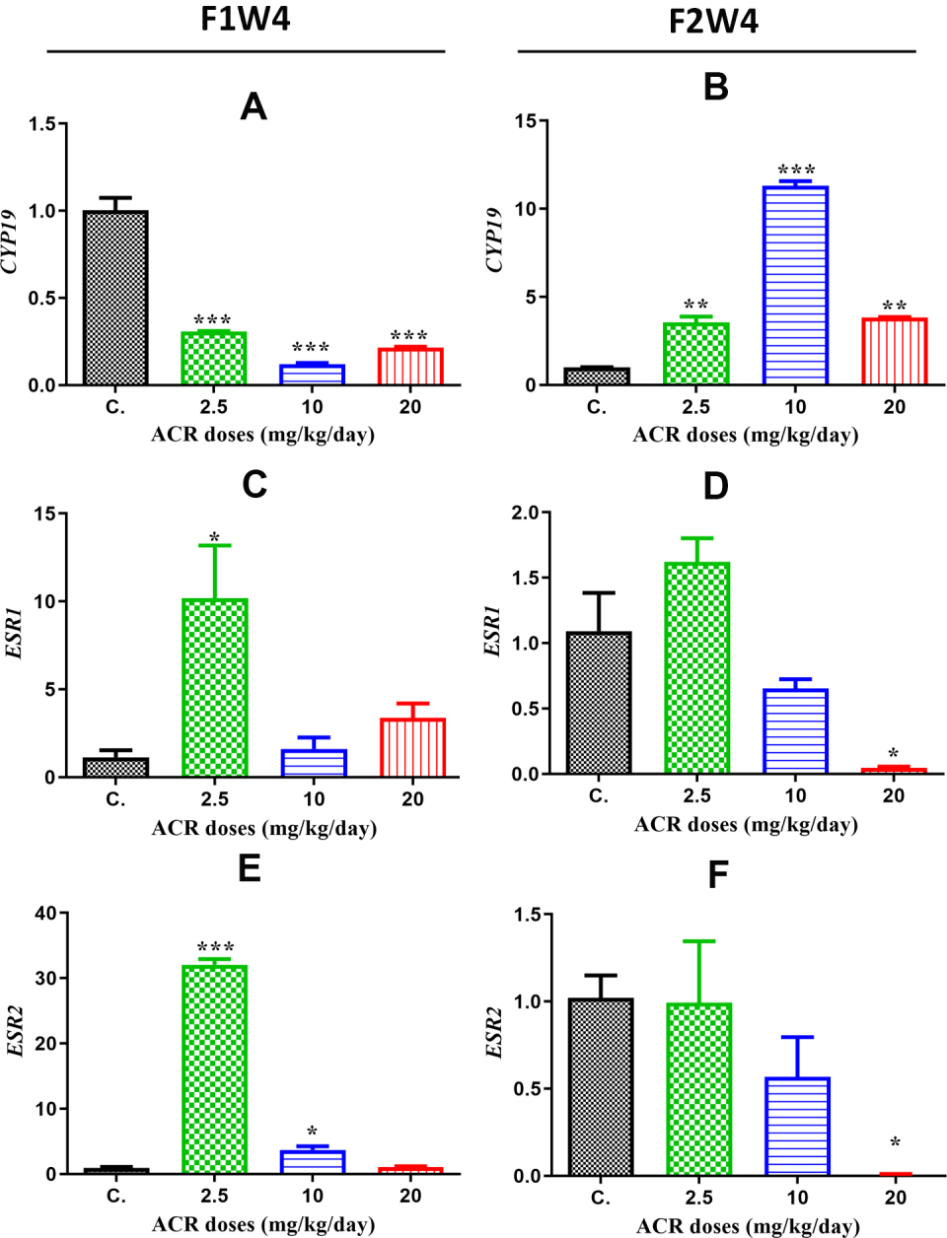
**mRNA expression levels of different genes in the ovaries of rats in the treatment groups compared to the control group from the different generations.** (**A**) *CYP19* mRNA levels in the first generation; (**B**) *CYP19* mRNA levels in the second generation; (**C**) *ESR*1 mRNA levels in the first generation; (**D**) *ESR*1 mRNA levels in the second generation; (**E**) *ESR*2 mRNA levels in the first generation; (**F**) *ESR*2 mRNA levels in the first generation. Values are means ± S.E.M. (*) P < 0.05; (***) P < 0.001.

*CYP19* mRNA levels were significantly decreased in the ovaries of all groups of AF1 (2.5, 10, and 20 mg/kg/day) compared to those of controls CF1 (Figure 7A). However, these *CYP19* mRNA levels were significantly increased in all groups of AF2 compared to the control group CF2 (Figure 7B).

### ESR1 gene

The *ESR1* levels in AF1 were significantly increased only in the rats exposed to the dose of 2.5 mg/kg/day compared to those in the control group (Figure 7C). However, the *ESR1* mRNA levels in AF2 significantly decreased with the dose of 20 mg/kg/day compared those in the control CF2 group (Figure 7D).

### ESR2 gene

Real-time PCR results showed that ACR treatment upregulated the mRNA expression of *ESR2* gene in AF1 with both doses of 2.5 and 10 mg/kg/day (Figure 7E) but downregulated it in AF2 with the dose of 20 mg/kg/day (Figure 7F).

## DISCUSSION

The research community has been concerned about the influence of environmental compounds on fetal or neonatal development since the perinatal phase is a time of considerable weakness for fetal and neonatal growth [32]. In fact, during pregnancy and lactation, the use of medications or toxic substances remains a public health issue, since it can damage the growth of some fetal organs and/or structures, including the ovary. During the embryonic stage or childhood, adverse external stimuli may result in life-long programming, leading to changes in tissue and/or organ function or gene expression during the developmental stage. From puberty to maturity, these changes can be preserved and even extended to the next generations and can affect future generations’ health [33, 34]. Particularly, EDCs are abundant globally, exposing humans to them on a daily basis. The endocrine system is extremely sensitive to EDCs, which interfere with metabolism, growth, and reproduction at various stages of life, particularly during the embryonic and pubertal stages. Many EDCs are known to target the female reproductive system, especially the ovary, which is considered the most important organ in a woman’s reproductive and endocrine processes [35]. Many reproductive health issues have been linked to EDC exposure, including miscarriage, premature ovarian failure, and elevated sex steroid hormone levels. Some EDCs and their effects on adult ovarian function have been widely studied over time [36]. Among the EDCs, ACR is a potential toxicant whose primary source is fried, baked, and roasted foods, which are generally, consumed by infants, adolescents, and adults worldwide [26, 37–40]. In our current study, the focus was on the effect of ACR during pregnancy on the ovarian function extended over two successive generations as the ovaries are considered one of the most sensitive organs to toxic substances and exposure during the fetal stage. It may pass through generations and affect the efficiency of reproductive organs [41, 42]. Our results indicated that ACR induced a significant increase in the weight of the ovaries from AF1 compared to that in the control group. This increase in ovary weight is consistent with previous studies that demonstrated that the treatment of pregnant female rats with some pollutants, such as sodium fluoride (NaF) and BPA, led to an increase in maternal ovary weight [43, 44]. Similarly, in utero exposure to nonylphenol (NP) caused an increase in offspring ovarian weight in mice [45]. It is known that ovarian weight in the normal rat does not show any significant changes although cyclic fluctuations are related to ovarian function throughout the estrous cycle. Thus, any increase or decrease in ovarian weight should be considered as an indication of a disruption in the ovarian function that could be associated with some disorders, such as reproductive aging, oocyte and follicle depletion, persistent polycystic ovaries, and luteal cyst development. The latter has been described in spontaneously cycling women with unexplained infertility [46]. In fact, the dominant luteinized follicle in a normal cycle shrinks and ruptures, but in some cases, its cystic nature can persist during the luteal phase, and it is defined as the “luteinized unruptured follicle syndrome” [47, 48]. Our examination to the histoarchitecture of the ovary showed that the different ACR doses induced follicle degeneration, degradation and vacuolation of the oocyte, and pyknosis of the nuclei in granulosa cells. Moreover, it has induced the appearance of cysts among the ovaries of different treated groups, which may be interpreted as luteinized unruptured follicle syndrome [48]. The formation of these cysts may be the cause of the increase in ovarian weight in AF1 female’s offspring, since follicle rupture is accompanied by a release of the accumulated follicular fluid, thus decreasing ovarian weight after ovulation. A similar previous study consistent with our result reported that the administration of aspirin to cycling mice increased ovarian weight, which appeared enlarged and contained cystic structures [49]. It has been reported that treatments of female rats with dihydrotestosterone and letrozole induced all ovarian and metabolic characteristics of PCOS [50].

With an increase in cystic structures, we noted an increase in plasma progesterone levels among all treated groups of AF1 compared to the control group. A very recent study presented the role played by progesterone in inhibiting granular cell proliferation and antral follicle growth using PI3K/AKT and MAPK pathways [51], which is consistent with our results. Moreover, reduced plasma estradiol and IGF-1 levels among all treated groups of AF1 compared to the control group has also previously been correlated with reduced fertility [52]. Similarly, the CYP19, which is responsible for synthesis of the estradiol hormone, has also decreased, accordingly illustrated by the reduction of mRNA *CYP19* gene expression detected by RT-PCR and CYP19 protein level detected by immunofluorescence. Since these hormones play a key role in female reproduction [53], their decline has undoubtedly negatively affected the fertility of females of the first generation. The TUNEL assay study confirmed that ovarian tissues, mainly stroma cells, have been strongly affected by ACR treatment with all doses although GDF-9 protein has been negatively affected only at a high dose (20 mg/kg/day). Numerous previous studies described the damage caused by EDCs on ovarian function. Among these studies, exposure to BPA decreased IGF1 and aromatase expression, and estradiol secretion in a dose-dependent manner in human granulosa cells [54] and affected steroidogenesis in cultured mouse preantral follicles [55, 56]. The reduced estradiol level was also described in mice offspring’s ovary under the effect of the heavy metal hexavalent chromium (CrVI), as a consequence of increased follicle atresia and apoptosis in oocytes and granulosa cells [57].

Interestingly, when comparing the results of the second generation to the first one, we realized that the effects of ACR exposure were not the same in each generation. In fact, we unexpectedly found that prenatal ACR exposure of AF2 decreased their ovarian weights, whereas it increased estradiol level, *CYP19* mRNA levels, and CYP19 protein expression in all treated groups. Particularly, IGF-1 levels have significantly increased only for the dose of 10 mg/kg/day. While the decreased ovarian weight in AF2 was consistent to previous studies that showed ACR effect on ovary weight by reducing it [29, 30, 35, 58, 59] and the decrease in ovary weight exhibited in the second generation of offspring females after neonatal exposure to NP [60], we did not expect the increase in levels of estradiol and its converter, the aromatase enzyme, at a time in which the ovary weight decreased. Indeed, the histopathological ovarian damages caused by ACR exhibited by the oocyte vacuolization and high number of ovarian apoptotic cells detected by TUNEL assay would not imply that sex steroid hormone levels should be spectacularly increased but decreased since growing follicles, mainly antral, are the primary producers of 17β-estradiol [61]. In agreement with this result, prenatal exposure to metformin increased the plasma estradiol levels in the adult offspring females, and this increase might be related to programming in theca and/or granulosa cells during development [62]. It has been described that high estrogen levels in females are associated with ovarian aging. In fact, treatment with doses of synthetic estrogens that has been used by adolescent girls [63] to reduce their height increased the risk of subfertility in later life as evidenced by accelerated ovarian aging with concomitant follicle pool depletion [64]. Moreover, the increased progesterone level in all treated groups of AF2 confirms this hypothesis since previous studies have shown that elevated progesterone levels are associated with reduced chances of pregnancy, which could be related to aging of the ovaries [65, 66]. Altogether, the present study suggests that the in utero multigenerational exposure to ACR highly reduced fertility and ovarian function in females of the first generation, while it has induced early ovarian aging in females of the second generation. The increased steroid hormone levels in females of the second generation may occur via different mechanisms, such as epigenetic changes, which might be the cause of physiological changes in the ovaries of females of the second generation. In fact, AF1 females were exposed to ACR when they were developing pups in the uterus of their mothers, while their germ cells that will form AF2 females were developing during this pregnancy. A similar study on zebrafish reported that the embryonic short-term exposure to the polycyclic aromatic hydrocarbon benzo (a) pyrene disrupted ovarian development in the ovary of adult females [67]. The disruption was mainly caused by high methylation levels in the GnRH and those of the GnRH receptor in the adult brain, which might cause the downregulation of the mRNA levels of *GnRH* and *GnRH* receptor genes. Similarly, we speculate that ACR mainly affected *CYP19a* gene during in utero second exposure by decreasing the methylation level of its promotor, which might cause the upregulation of the *CYP19* mRNA levels and its protein, leading to an increase in estradiol level in AF2. Although we did not analyze the methylation of *CYP19a* gene, a very recent study by [68] strongly supports our hypothesis. In fact, the authors of this study concluded that the environment experienced by mothers can affect their offspring sex ratio via environment-induced DNA methylation changes in the gonads. They found a very strong correlation between the methylation state of *CYP19a* gene in the female gonads of zebrafish and the sex ration of the entire population. Indeed, an increase in the methylation level of *CYP19a1a* gene was associated with heat, leading to masculinization of the whole population, while cadmium (Cd) influenced the methylation levels of foxl2a/dmrt1, resulting in the progressive feminization of the offspring over generations [68].

## CONCLUSIONS

To the best of our knowledge, this is the first study to examine the effects of the multigenerational exposure to ACR on ovarian function and fertility in female rat offspring. In this study, we found that the first generation reacted differently from the second generation. Indeed, maternal exposure to ACR caused an ovarian disruption in AF1 as evidenced by severe histopathological damage, development of cysts, and high apoptosis in the stroma cells, and decreased plasmatic estradiol levels and its corresponding CYP19 gene and protein expression. However, it has induced early ovarian aging in AF2 characterized by high estradiol and progesterone levels, upregulation of CYP19, and apoptotic cell death in the stroma. Moreover, this study provides some interesting evidence for the eventual implication of the epigenetic impacts of endocrine disruptors on female reproduction across generations. Future studies, using genome wide DNA methylation approaches for some specific key biomarkers of ovarian development, such as CYP19, are fundamental to determine how prenatal exposure to endocrine disruptors could drive adverse secondary phenotypic effects among the future generations in both humans and animals.

## MATERIALS AND METHODS

### Study design and sampling

Twenty healthy pubertal virgin female Wistar-Albino rats (200–250 g and at age of 8 weeks) were weighed, housed individually in cages, and maintained in a facility with a diet of standard laboratory chow and a 12 h light:12 h darkness photoperiod at a temperature of 21 ± 1° C. The appearance of a white vaginal plug on the floors of cages indicates that the mating was successful, and that day was considered as day 0 of gestation (GD 0). The pregnant females were divided into four groups, and the treatment was started at GD 6 and continued until GD 21 of gestation as follows:

1) The first group of females were considered as the control group and were gavaged with distilled water (n = 5). The second group of females (n = 5) received an ACR by oral treatment at a dose of 2.5 mg/kg. The third group of females (n = 5) received an ACR by oral treatment at a dose of 10 mg/kg. The fourth group of females (n = 5) received an ACR by oral treatment at a dose of 20 mg/kg [69]. After parturition, we obtained the first generation of offspring obtained from ACR-treated mothers and therefore are called animals of the first generation (AF1), and those obtained from the control group are called control group of the first generation (CF1). A proportion of the AF1 and CF1 offspring females were anesthetized when they reach 4 weeks of age (before puberty). For euthanasia, they were transferred individually to a transparent plastic box connected to a carbon dioxide tube at a flow rate of 10 L/h for 10 min. Blood samples were drawn from the heart and transferred directly to tubes containing an anticoagulant (EDTA) to obtain the plasma after centrifugation. The ovaries were cleaned, quickly measured, and labelled according to their origin (groups). The remainders of AF1 and CF1 females were allowed to reach sexual maturity and mated with males and treated exactly as their mothers. After parturition of AF1 and CF1 females, the second generation of offspring was obtained from ACR-treated AF1 mothers and called ACR offspring of the second generation (AF2) and those obtained from the control group are called control of the second generation (CF2). The AF2 and CF2 offspring females were treated exactly the same as those of their mothers and grandmothers. Briefly, the females were anesthetized when they reached 4 weeks of age (before puberty). The blood and ovaries were collected, labeled according to their origin (groups), and fixed.

### Histological preparation

Ovary samples that were fixed in 10% neutral buffered formalin (NBF) for 24 h, embedded in paraffin and cut in sections with a thickness of 5–7 μm, collected on a hotplate and transferred to glass slides containing warm (30° C) water and albumin glycerol fixative for adhesion. Wrinkles were removed, and the sections were stained with hematoxylin and eosin.

### Plasma hormone concentration assay

The concentrations of progesterone, testosterone, estradiol and IGFI were measured by using ELISA kits from DSL (Webster, TX, USA) according to the manufacturer's instructions.

### Immunofluorescence staining and confocal microscopy

The immunostaining was investigated as described in our previous studies [70, 71]. Slides containing tissue sections were placed on hotplate (60° C) and dewaxed with xylene. Then, they were rehydrated and washed twice with distilled water and 3 times with 1x PBS. The slides were removed from washing process and dried. After drying, the slices were placed in a suitable container and the tissue sections were permeabilized using 0.1% Triton X-100 with 0.1% sodium citrate, and treated with blocking buffer (1% BSA in PBS) at RT. The slides were placed into the humid box and treated with the primary antibody solution (CYP19 and GDF-9) (dilution 1:100) from Co., Ltd. (China), overnight at 4° C on a flat balanced surface in the dark. Negative control slides consisted of sections incubated with PBS (Supplementary Figure 1). After being washed four times with 1X PBS, slides were treated with the secondary antibody, antibody (FITC) (dilution 1:2000, Abcam, USA) 45 min at room temperature (RT) in the dark. Slides were washed with PBS and then TE buffer before adding Hoechst solution (dilute 1:15000, Hoechst 33342, Life Technologies, USA). Finally, the sections were mounted in 50% glycerol/TE solution, and the edges were covered with nail polish. Sections were observed and imaged for signal quantification with a spinning disk confocal microscope from Zeiss. The signal intensity for protein expression was analyzed by Zen 3.1 service (ZEN lite) and quantified using GraphPad Prism 9 program (GraphPad Software).

### Apoptosis analysis by TUNEL assay

The apoptotic analysis was investigated as described in our previous studies [35, 70, 72]. Briefly, the ovaries fixed in NBF for 24 h were preserved in 70% alcohol. Paraffin sections with a thickness of 3 μm were prepared and mounted on coated slides at RT. The slides were deparaffinized, rehydrated, and washed in PBS. Then, the tissue sections were incubated with a Proteinase K working solution at 37° C for 15–30 min and permeabilized in 0.1% Triton X-100 with 0.1% sodium citrate. TUNEL staining was conducted following the manufacturer’s instructions of an *In Situ* Cell Death Detection Kit, TMR red 12156792910 (Roche Diagnostics, Mannheim, Germany). The positive control was incubated in the same conditions as the rest of the samples but after pretreatment with recombinant DNase I for 10 min before the reaction mixture (Supplementary Figure 2). Hoechst was used to stain the nuclei, and the quantification of the signal for TMR-red was analysed by Zen 3.1 (ZEN lite), and statistical significance was tested with GraphPad Prism 9 (GraphPad Software).

### Analysis of gene expression

RNA was isolated using the RNeasy Mini Kit (Qiagen, Westburg, The Netherlands) with DNase treatment on columns using an RNase-free DNase kit (Qiagen). The quality and integrity of the extracted RNA were verified by measuring the 260/280 nm ratio using a Nanodrop. cDNA was then reverse-transcribed from 0.1 to 0.5ug total RNA using RT–PCR and primer sets using an iScript ™ cDNA synthesis kit (Applied Biosystem, Carlsbad, CA, USA) according to the manufacturer’s instructions. Real-time PCR (RT–PCR) was performed using SYBR green and an applied biosynthesis 7500 Fast RT–PCR system (Carlsbad, CA, USA) with the gene-specific primers shown in Table 1. For each gene transcript, the relative amount was calculated using the 2^-ΔΔCT^ and normalized by referencing to the gene GAPDH.

**Table 1:** Primers for real-time RT-PCR.

**Gene symbol**		**Sequences**
***CYP19A***	Forward:	G G A G A A T T C A T G C G A G T C T G G
	Reverse:	T G C C G A A T C G A G A G C T G T A A
***ESR1***	Forward:	C A T C G A T A A G A A C C G G A G G A
	Reverse:	A A G G T T G G C A G C T C T C A T G T
***ESR2***	Forward:	G A A G C T G A A C C A C C C A A T G T
	Reverse:	C A G T C C C A C C A T T A G C A C C T

### Statistical analysis

Data analysis was conducted using GraphPad Prism version 9. One-way analysis of variance, followed by Tukey’s multiple comparison, was used for statistical comparisons. All values are presented as mean ± standard deviation (SD). Significance was set at a P-value < 0.05.

### Data availability statement

The data that support the findings of this study are available from the corresponding author (Abdel Halim Harrath) upon reasonable request.

## Supplementary Material

Supplementary Figures
