# Supplementary material for: Fetal programming: in utero exposure to acrylamide leads to intergenerational disrupted ovarian function and accelerated ovarian aging
Source: Aging (Albany NY). 2022 Sep 6;14(17):6887–904. doi: 10.18632/aging.204269 (PMC9512500; doi:10.18632/aging.204269)
Supplement: Supplementary Figures [file aging-14-204269-s001.pdf]

SUPPLEMENTARY FIGURES

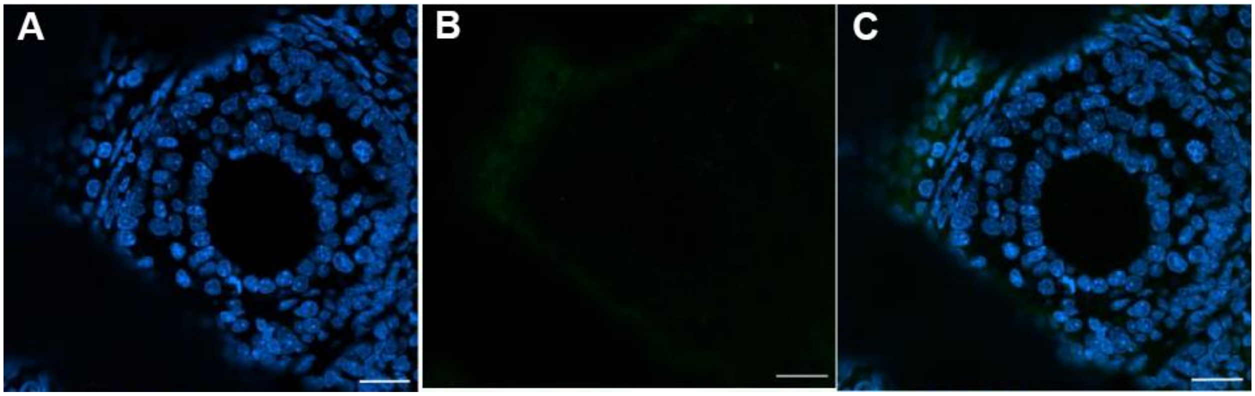

Supplementary Figure 1. The ovarian CYP19 localization and expression in 4-week-old females of negative control. (**A**) stained with Hoechst, (**B**) stained with FITC conjugated secondary antibody, and primary antibody was replaced with PBS, (**C**) merged image stained with Hoechst and FITC.

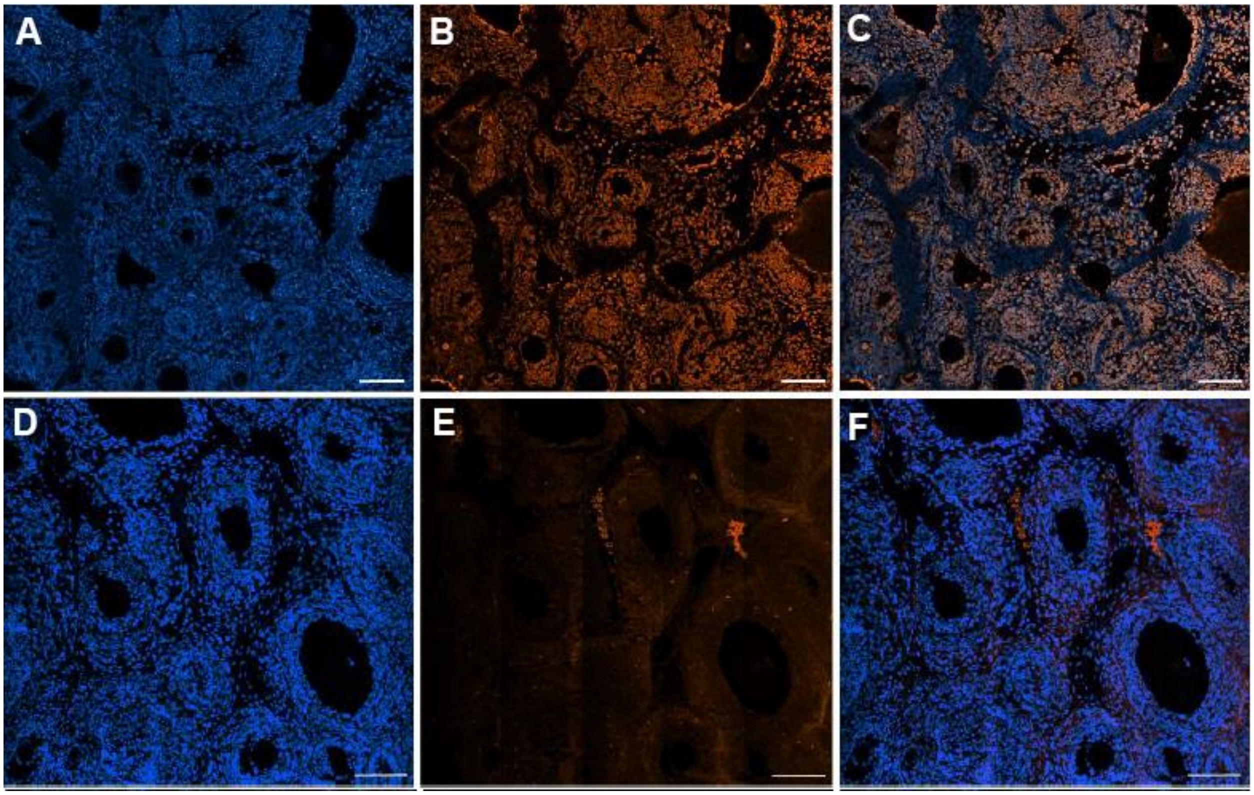

Supplementary Figure 2. The TUNEL reaction in 4-week-old females of positive control (**A**–**C**), and negative control (**D**–**F**).
